# Antitumor Potential of *Lippia citriodora* Essential Oil in Breast Tumor-Bearing Mice

**DOI:** 10.3390/antiox10060875

**Published:** 2021-05-30

**Authors:** Katerina Spyridopoulou, Tamara Aravidou, Evangeli Lampri, Eleni Effraimidou, Aglaia Pappa, Katerina Chlichlia

**Affiliations:** 1 Department of Molecular Biology and Genetics, Democritus University of Thrace, University Campus-Dragana, 68100 Alexandroupolis, Greece; aikspiridopoulou@gmail.com (K.S.); tamara.arav@gmail.com (T.A.); evangeli.lampri@gmail.com (E.L.); apappa@mbg.duth.gr (A.P.); 2 Department of Medicine, Democritus University of Thrace, University Campus-Dragana, 68100 Alexandroupolis, Greece; eeffraem@med.duth.gr

**Keywords:** *Lippia citriodora*, essential oil, antiproliferative effect, antimigratory activity, breast cancer, oral administration

## Abstract

*Lippia citriodora* is a flowering plant cultivated for its lemon-scented leaves and used in folk medicine for the preparation of tea for the alleviation of symptoms of gastrointestinal disorders, cold, and asthma. The oil extracted from the plant leaves was shown to possess antioxidant potential and to exert antiproliferative activity against breast cancer. The aim of this study was to further investigate potential antitumor effects of *L. citriodora* oil (LCO) on breast cancer. The in vitro antiproliferative activity of LCO was examined against murine DA3 breast cancer cells by the sulforhodamine B assay. We further explored the LCO’s pro-apoptotic potential with the Annexin-PI method. The LCO’s anti-migratory effect was assessed by the wound-healing assay. LCO was found to inhibit the growth of DA3 cells in vitro, attenuate their migration, and induce apoptosis. Finally, oral administration of LCO for 14 days in mice inhibited by 55% the size of developing tumors in the DA3 murine tumor model. Noteworthy, in the tumor tissue of LCO-treated mice the apoptotic marker cleaved caspase-3 was elevated, while a reduced protein expression of survivin was observed. These results indicate that LCO, as a source of bioactive compounds, has a very interesting nutraceutical potential.

## 1. Introduction

Being stationary autotrophs, plants need to overcome a variety of challenges such as herbivores, pathogens, and the need for a steady flow of nutrients despite the environmental changes. In order to cope with the various stresses and damages induced by a dynamic environment, plants have evolved to synthesize secondary metabolites that allow them to interact with their environment [1,2]. These low-molecular-weight substances, which can be species- or genera-specific [2], represent a huge and heterogeneous group of bioactive compounds, used as high-value chemicals [3].

Plant secondary metabolites are a unique source for pharmaceuticals [4], insecticides [5], flavoring agents, dyes, etc. [3]. Noteworthy, various bioactivities were identified in different phytochemicals such as antibacterial [6], antiviral [7], antifungal [8], antioxidant [9], and anti-inflammatory [10] activities. Most importantly, several plant extracts exert significant antitumor effects, namely, antiproliferative, redox regulating, cell cycle arrest-inducing, anti-migratory, and pro-apoptotic [11,12,13,14]. Carcinogenesis and tumor growth are complex processes that involve various mechanisms and the recruitment of a diverse range of molecules. Phytochemicals, being functionally pleiotropic, may lead to simultaneous targeting of the various events/pathways involved in cancer development and progression [15]. Certain dietary phytochemicals, besides their direct antitumor effects, were shown to enhance the therapeutic potency of chemotherapeutic agents [16]. Crucially, the anticancer properties of various phytochemicals were linked to their antioxidant activity [17].

*Lippia citriodora* is a flowering subshrub/shrub, native in South America, that was introduced in Europe at the end of 17th century [18]. The plant is also known as *Lippia triphylla*, *Verbena triphylla*, *Aloysia triphylla*, *Aloysia citriodora*, *Verbena citriodora,* or *Lemon verbena*. *L. citriodora* is mostly cultivated for its lemon-scented leaves, traditionally used in folk medicine for the alleviation of gastrointestinal symptoms, fever, cold, asthma, diabetes, spasms, insomnia, and anxiety [19,20]. Plant extracts were shown to exert significant antioxidant activity that was associated with their flavonoid and phenolic content [21,22,23,24]. Antioxidant properties of *L. citriodora* were evaluated in various in vitro, ex vivo, or in vivo systems, employing different biochemical assays. *L. citriodora* infusion was shown to act as a free radical scavenger against superoxide, hydroxyl radicals, and hypochlorous acid [23]. Aqueous extracts of the plant were shown to inhibit the ABTS (2,2′-azino-bis(3-ethylbenzothiazoline-6-sulphonic acid)) and DPPH (2,2-Diphenyl-1-picrylhydrazyl) radicals and protect against protein carbonylation and lipid peroxidation in Wistar rat brain homogenates [25], while alcoholic and aqueous extracts, upon oral administration, were shown to elevate the blood glutathione levels in the alloxan-induced diabetic rat model [26]. Noteworthy, both the essential oil investigated in this study as well as citral, a mixture of two terpenoid isomers that was identified as the oil’s main constituent, were shown to inhibit the H_2_O_2_-induced oxidative damage in human Jurkat T-leukemia cells [27]. Besides the plant’s well-documented antioxidant potential, recent studies demonstrate that various extracts from *L. citriodora* have the potential to inhibit the growth of cancer cells in vitro [27,28,29,30]. Moreover, citral, the main constituent of the essential oil from *L. citriodora* [27], exerts antitumor activity in murine breast cancer models upon oral administration [31,32].

Breast cancer is the most frequent cancer and the leading cause of cancer death in women [33]. Because of the heterogeneity of breast cancer as a disease, treatment protocols and prognosis vary greatly between different patients [34]. Several important risk factors for breast cancer such as menopause, aging, genetic predisposition or estrogens, have been associated with oxidative stress [35]. Consecutively, it has been proposed that compounds with antioxidant potential such as the various extracts from *L. citriodora* discussed above could prevent certain biochemical processes that lead to breast cancer development [36].

Therefore, the aim of this work was to examine the potential antitumor effects against breast cancer of the essential oil extracted from the leaves of *L. citriodora* (LCO) as a plant-specific mixture of various bioactive compounds. Specifically, we assayed the potential growth inhibitory, anti-migratory, and pro-apoptotic activities of the essential oil in vitro. Moreover, we examined whether oral administration of the oil attenuates growth of breast cancer in vivo in the experimental DA3 mouse tumor model.

## 2. Materials and Methods

### 2.1. Plant Material and Essential Oil Extraction

Collection and identification of plant material was described in a previous study by our team regarding the biological properties of *Lippia citriodora* oil [27]. Briefly, plant samples of small shrubs were purchased by Vioryl S.A. from an herbal market (Afidnes, Athens, Greece), identified as *Lippia citriodora* by a botanist, and planted and maintained in pots until their first inflorescence in May/June when the leaves and stems were collected. The collected parts of the herb were subjected to hydrodistillation in a laboratory-scale, water-steam distillation apparatus as described in our team’s previously published study [27]. The composition of the extracted oil was analyzed by Gas chromatography–mass spectrometry (GC-MS) (GC: 6890 A, Agilent Technologies, USA; MSD: 5973, Agilent Technologies, Santa Clara, CA, USA) using an HP-1 ms column (25 m, 0.2 mm i.d., 0.33 μm film thickness). The detailed analysis process and chemical composition of the oil are described in Fitsiou et al. [27].

### 2.2. Cell Line

DA3 cells is a well-characterized tumor cell line that was derived from the D1-DMBA-3 mammary tumor, syngeneic to BALB/c mice [37]. The cells were maintained under sterile conditions at 37 °C in a humidified atmosphere of 5% CO_2_ in DMEM supplemented with 10% fetal bovine serum, penicillin (100 U/mL), and streptomycin (100 μg/mL).

### 2.3. Animals

Mice (BALB/c, female, *n* = 18) that were 6–8 weeks old and weighted 20–25 g, were purchased from the Animal Facility of Pasteur Institute (Athens, Greece). Animals were housed in polycarbonate cages (maximum of 5 mice per cage) and maintained at room temperature, on a 12 h light-12 h dark cycle at the Animal House of Medical School at the University of Ioannina (Greece). Tap water and a commercial, pelleted diet (Mucedola) were provided *ad libitum* to all animals. Animal experiments were approved by the Animal Care and Use Committee of the Veterinary Department of Ioannina Prefecture (license number EL20BIO02). All animal procedures were carried out in accordance with the principle of the 3 Rs (replacement, refinement, reduction) and all mice used were not subjected to any pain or discomfort.

### 2.4. DA3 Experimental Tumor Model

Mice were separated into two independent groups (nine mice per group) and a previously described treatment protocol was followed [9,11,12,38,39]. Briefly, LCO was administered *per os* by oral gavage at a daily dose of 0.552 g/kg of animal body weight diluted in a final volume of 150 μL of corn oil (vehicle), for 14 days. Animals in the control group received an equal volume of plain corn oil. Mice were monitored daily for weight fluctuations and signs of discomfort or disease. DA3 cells (5 × 10^6^) were injected subcutaneously, in the scruff of mice’s necks on the 12th day of LCO administration. Animals were euthanized by cervical dislocation on the 7th day post cancer cells inoculation. Developed tumors, spleens, and livers were surgically removed from all animals. The organs were weighed and tumor dimensions were defined by an electronic micrometer. Tumor volume was calculated by the modified ellipsoid formula (mm^3^):

*Tumor volume* = 0.5 × (*width*^2^ × *length*) (1)

### 2.5. Chemicals and Reagents

Acetic acid, dimethyl sulfoxide (DMSO), trichloroacetic acid (TCA), sulforhodamine B (SRB) and Trizma base were purchased from Sigma-Aldrich (St. Louis, MO, USA); Annexin V-PI kit was purchased from BD Biosciences (Franklin Lakes, NJ, USA); Dulbecco’s Modified Eagle’s Medium (DMEM) and phosphate-buffered saline (PBS) were purchased from Gibco (Thermo Fisher Scientific, Waltham, MA, USA); trypsin, fetal bovine serum (FBS) and penicillin/streptomycin, were purchased from Biosera (Boussens, France). Formalin and Hematoxylin were purchased from Merck (Merck Millipore, Billerica, MA, USA) and eosin, xylole and paraffin were purchased from Diapath (Martinengo BG, Italy). DAB (3,3’-Diaminobenzidine) was purchased from Dako (Agilent Technologies, Santa Clara, CA, USA). All antibodies were purchased from Cell Signaling (Danvers, MA, USA).

### 2.6. Cell Growth Assay

Cell growth rate was analyzed by the Sulforhodamine B (SRB) assay [40], as previously described [11]. Briefly, 8000 DA3 cells were seeded per well in a 96-well plate. Cells were exposed to a wide range of increasing LCO concentrations (0–0.92 mg/mL) dissolved in DMSO (1:1 *v/v*), for 24, 48, or 72 h. Control cells were incubated in DMSO-containing DMEM (DMSO concentration ≤ 0.1% *v/v*). After treatment, cells were fixed with TCA for 1 h and stained with SRB dissolved in acetic acid for 30 min. Tris base was added in SRB-stained cells in order to dissolve the SRB cell-bound stain before measuring the samples’ absorbance (optical density, OD) at 492 nm. For the OD measurement, a microplate reader (Enspire, Perkin Elmer) was employed. Results presented are representative of at least three independent experiments. The IC_50_ values were calculated using the Sigma Plot software v.11. The % inhibition of cell growth was calculated by the following formula:

% *growth* = (*mean OD sample*)/(*mean OD control* × 100) (2)

### 2.7. Wound-Healing Assay

DA3 cells were seeded in 12-well plates at a seeding density of 3 × 10^5^ cells/well. After 24 h, when a confluent monolayer was formed, a 10 μL pipette tip was used to scratch the monolayer across the well. Next, monolayers were washed with PBS so that the detached cells could be removed and either DMSO (DMSO concentration ≤ 0.1% *v/v*) for control cells or LCO-containing medium for treated cells was added to the wells. To exclude the growth inhibitory effect of LCO as a potential mechanism of wound-healing inhibition, non-toxic concentrations were used (as determined by the SRB assay) for the treatment of the cells. Specifically, cells were treated with 46 μg/mL of LCO. Cells were photographed with a light microscope (Zeiss, Göttingen, Germany) that was equipped with a digital camera. Multiple photographs per time point were analyzed with ImageJ software (NIH, Bethesda, MD, USA) from three independent experiments and the average % wound area (% open image area) was calculated.

### 2.8. Flow Cytometric Analysis of Apoptosis by Annexin V and Propidium Iodide Staining

Detection of apoptotic cells was performed using the Annexin V-PI double staining method [41]. A commercially available kit was used according to the manufacturer’s instructions. Briefly, cells were seeded in 6-well plates at a seeding density of 2 × 10^5^ cells/well. After an overnight incubation, cells were treated with 89 or 184 μg/mL of LCO for 24, 48, or 72 h. Cells treated with appropriate DMSO (vehicle) concentrations were used as control. Following collection, cells were stained and analyzed on an Attune NxT flow cytometer (Thermo Fisher Scientific, Waltham, MA, USA). Data obtained were analyzed using FlowJo V10 software (Tree Star, Inc., Ashland, OR, USA).

### 2.9. Immunohistochemical Analysis

Excised tumors were fixed in 10% formalin before being dehydrated in graded concentrations of ethanol, xylole, and embedded in paraffin. Paraffin tissue blocks were cut into 3 μm thick sections with a microtome. Next, the sections were mounted onto charged glass slides. An hematoxylin and eosin stained section was obtained from each tissue block. Immunostaining was performed based on streptavidin-biotin peroxidase labeled method. Deparaffinization and hydratation were done by immersing the tissue sections into graded concentrations of ethanol to deionized water. Primary antibodies, against survivin (#2808 Cell Signaling, dilution 1:50) or cleaved caspase 3 (#9661 Cell Signaling, dilution 1:1000), were then applied to the tissues and incubated overnight at 4 °C. Immunoreactivity was visualized with 3,3′-Diaminobenzidine (DAB) followed by counterstaining with hematoxylin. For negative controls, the primary antibody was omitted. An image analysis system composed of the Olympus BX43 upright microscope, digital camera Olympus Cam-SC30 (Olympus Europa, Hamburg, Germany) and soft analysis (analySISH) was used. Two observers selected at least 10 representative regions from all the sections of each case, where immunopositive tumor cells were counted by using the ×40 objective lens, adopting a continuous score system. The expression was defined as the percentage of positive cells in the total number of the counted cells (number of immunopositive cells/total number of the counted cells).

### 2.10. Statistical Analysis

Data are representative of at least three independent experiments and are presented as mean ± SD. For statistical analysis and graphing, the Sigma Plot v.11 software (Systat Software Inc., San José, CA, USA) was used unless stated otherwise. For statistical comparisons between groups Student’s *t*-test or Mann-Whitney test for non-parametric variables were performed. Differences between groups were considered significant when *p* < 0.05 (* *p* < 0.05, ** *p* < 0.01, *** *p* < 0.001).

## 3. Results and Discussion

### 3.1. Chemical Composition of LCO

Volatile profile analysis by GC-MS conducted by our team identified the composition of LCO, which was described in detail in a previous published study [27]. Briefly, 43 compounds representing the 87% of the total chromatographic area were identified. The two major compounds were determined to be geranial (*trans*-citral) and neral (*cis*-citral), reported as citral, which is the sum of the two isomers (Table 1). Neral and geranial accounted for 17.2% or 26.4%, respectively, of the total chromatographic area, which is in accordance with the literature where citral was previously reported as one of the main components of the oil extracted from plants of the citriodora species [18]. Noteworthy, the *trans*-isomer geranial which is 1.5 times more abundant in LCO than neral, was reported to exert more potent antitumor properties as compared to the *cis*-isomer, neral [42]. Interestingly, *L. citriodora* oil with a similar composition was previously shown to possess antioxidant properties [22], while our team investigated the antioxidant potential of LCO and reported its ability to protect Jurkat cells against H_2_O_2_-induced oxidative damage as well as LCO’s moderate direct antioxidant activity analyzed by the DPPH and ABTS assays [27].

### 3.2. Antiproliferative Activity of LCO

**Figure 1:**
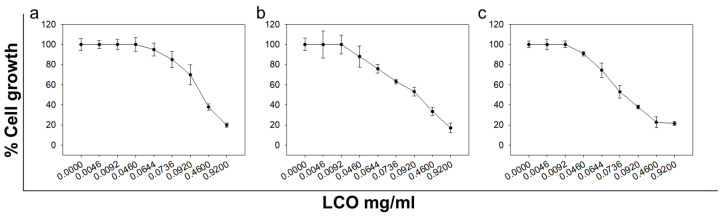
In vitro anticancer activity of LCO. Antiproliferative effect of increasing doses of LCO at (**a**) 24 h, (**b**) 48 h, or (**c**) 72 h on DA3 murine breast cancer cells. Percentage (%) of cell growth was analyzed by the SRB assay. All data shown are representative of at least three independent experiments. Values represent mean ± SD.

LCO exhibited a dose- and time-dependent growth inhibitory activity against DA3 murine mammary adenocarcinoma cells (Figure 1). The IC_50_ values for 24, 48, or 72 h were determined to be 96.4 ± 8.9, 77.8 ± 1.5, or 70.7 ± 5.5 μg/mL, respectively (Table 2). The higher IC_50_ values for 24 h compared to 48 and 72 h and for 48 h compared to 72 h implied that the growth inhibitory effect exerted by LCO against DA3 cells was time dependent. Cytotoxicity of LCO was previously reported by our team against human melanoma A375, hepatic HepG2, breast MCF-7, and colonic Caco2 cancer cell lines. We also observed a moderate cytotoxic activity of LCO against THP-1 human leukemic cells’ line (EC_50_ > 100 μg/mL) [27], which is in agreement with the results reported by Escobar et al. [28]. LCO was also shown to inhibit the growth of murine P815 mastocytoma [29], human breast adenocarcinoma MDA-MB-231 cells, as well as chronic myelogenous erythroleukemia K562 and neuroblastoma SH-SY5Y cells [30]. Besides the essential oil extracted from the plant, various other *L. citriodora* extracts were reported to exert antiproliferative activity against cancer cell lines as well [43,44]. Noteworthy, *L. citriodora* extracts appear to exert cancer-specific, growth-inhibitory effects. Escobar et al. described that LCO did not significantly affect the proliferation rate of normal Vero monkey kidney cells [28]. The potential selective antiproliferative activity of *L. citriodora* extracts against cancer cells was also indicated by the cytotoxic effect of the herbal substance acteoside, isolated from *L. citriodora* leaves, against A5 murine metastatic spindle carcinoma cells. Interestingly, acteoside did not affect to the same extent the viability of C5N normal mouse keratinocytes [45]. Moreover, LCO exhibited moderate antiproliferative activity against human normal ESCs (endometrial stromal cells) [30]. Thus, even though there are only a few studies investigating the in vitro antiproliferative potential of LCO, these studies suggest that the oil extracted from *L. citriodora* exerts potent, diverse, and selective antitumor activities.

### 3.3. Evaluation of the In Vitro Pro-Apoptotic Potential of LCO

**Figure 2:**
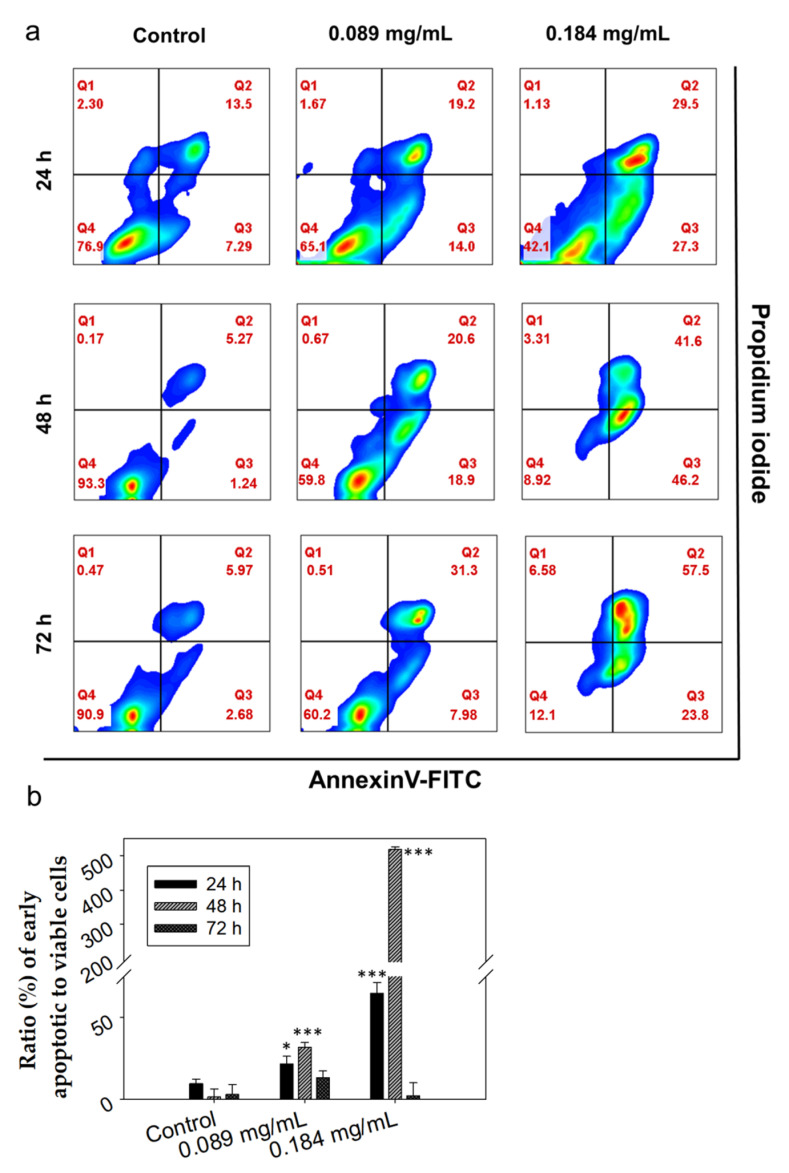
LCO induces apoptotic cell death in DA3 murine breast adenocarcinoma cells detected by flow cytometry. Cells were treated with 0.089 or 0.184 mg/mL of the LCO for 24, 48, or 72 h and stained with Annexin V-FITC and PI before being analyzed by flow cytometry. (**a**) Density plots of control and treated cells. The percentages of viable (Annexin V-FITC and PI negative), early apoptotic (Annexin V-positive and PI-negative), and late apoptotic/necrotic (Annexin V/PI double-positive) cells are indicated on the plots. (**b**) Percentage (%) ratio of early apoptotic to viable cells. At least 10,000 cells were analyzed per sample. Results are presented as the mean ± SD of three independent experiments. Asterisks indicate statistically significant difference between control and treated cells (* *p* < 0.05, *** *p* < 0.001 Student’s *t*-test).

The interesting observation of the time-dependent, growth inhibitory effect of LCO led us to further investigate the potential mechanism involved. Interestingly, it has been reported in the literature that both the oil as a mixture [29] and acteoside, an herbal substance isolated from *L. citriodora* [45], may induce apoptosis in cancer cells. Moreover, citral, which has been identified as the major component of LCO [27], was reported to induce apoptosis in breast cancer cells in vitro and in vivo in murine tumor models [31,46]. Thus, we investigated the potential pro-apoptotic activity of LCO against DA3 cells by the flow cytometric analysis of Annexin V and propidium iodide (PI) double staining method for the identification of early apoptotic cells. Our results are in accordance with the literature and indicate that LCO induces morphological changes in cells that are typical during apoptosis in DA3 cells. Specifically, cells treated with 0.089 or 0.184 mg/mL of LCO for 24 h exhibited, compared to control, a 12% or 55% rise, respectively, in the ratio of early apoptotic to viable cells (Figure 2). Cells treated for 48 h exhibited an increase of 30% or 517%, while cells treated for 72 h exhibited an increase of 10% or a decrease of 1% (as progressively, an increasing number of cells undergoes cell death) in the ratio of early apoptotic to viable cells for 0.089 or 0.184 mg/mL of LCO, respectively.

This observation indicates that LCO induces concentration-dependent apoptotic effects in DA3 cells.

Apoptosis is a mechanism that comprises a very significant target for anticancer therapy [47]. There have been previous reports on the pro-apoptotic activity of essential oils extracted from different plant species against various cancer cell lines [11,48,49]. These extracts, due to their diverse chemical composition, comprise a valuable source of bioactive compounds [50]. Our results indicate that LCO could be a good candidate for the identification of apoptosis-promoting phytochemicals.

### 3.4. LCO Exerts Antimigratory Effects

**Figure 3:**
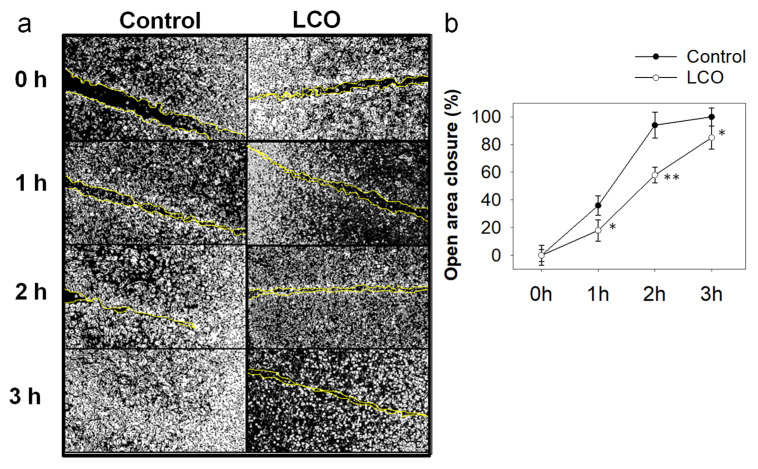
Effect of LCO on migration of cancer cells. Wound-healing assay for DA3 cells treated with 46 μg/mL LCO or DMSO for control. Migration rate of cells was monitored with an optical microscope equipped with a digital camera. (**a**) Photographs of the wounds captured at the indicated time points. (**b**) Quantification of the percentage of wound closure was estimated by ImageJ software analysis. Data are presented as the mean ± SD of three independent experiments. Asterisks indicate statistically significant differences (* *p* < 0.05, ** *p* < 0.005, Student’s *t*-test).

Furthermore, we investigated whether LCO affects migration of DA3 cells in vitro. Migration of cancer cells is a crucial step in cancer invasion and metastasis [51]. Identification of novel therapeutic compounds that have the potential to attenuate cancer cell migration could lead to the development of novel or combination anticancer strategies. Noteworthy, various phytochemicals have been reported to block migration of cancer cells in vitro [11,12,52]. Despite the lack of studies regarding the potential antimigratory effect of LCO, citral, the most abundant phytochemical in LCO, was shown to inhibit the migration of both human and murine breast cancer cells [32,53]. Thus, we employed the wound-healing assay in order to investigate whether LCO attenuates migration of DA3 cells. Noteworthy, the cells were treated with a non-toxic concentration of LCO (46 μg/mL) that, according to our SRB results (Figure 1), did not significantly affect cell growth. Our results indicate that it took more time for the LCO-treated cells to fill the open area compared to control cells (Figure 3). Specifically, the mean ratio of open area closure in control cells reached 94% after only 2 h, while the LCO-treated group exhibited only a 58% of open-area-closure ratio in the same time point. Wound closure occurred after 3 h in control cells while the closure was not complete in LCO-treated cells that exhibited a remaining 15% of open image area. Considering the reported antimigratory activity of citral, LCO’s observed migration-attenuating effect might be attributed to its major constituent.

Thus, we suggest that a comparative study regarding the antiproliferative and antimigratory activities of citral and LCO be carried out in order to investigate whether citral or a combination of other phytochemicals present in LCO exert the biological reactivity of the oil.

### 3.5. Oral Administration of LCO Attenuates Tumor Growth in DA3 Breast Cancer Model in Mice

**Figure 4:**
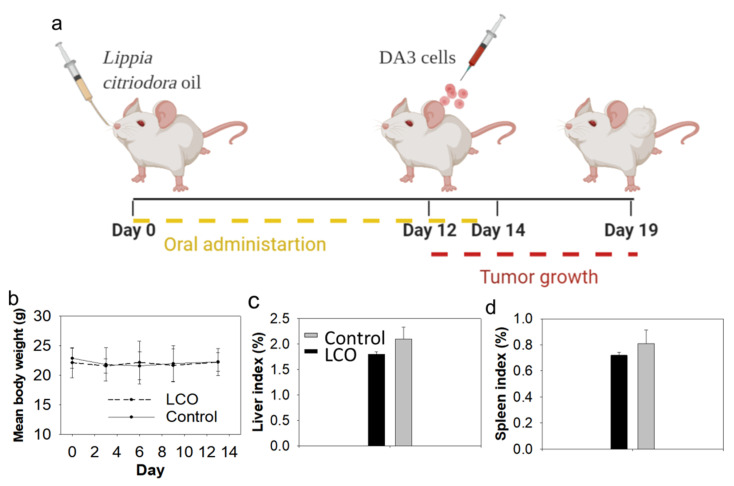
Orally administered LCO in mice is well tolerated. (**a**) LCO was administered daily to BALB/c mice by oral gavage for 14 days (0.552 g/kg body weight/day). On the 12th day, mice were inoculated subcutaneously with DA3 cancer cells (5 × 10^6^ cells per mouse) and, 7 days later, animals were euthanized. (**b**) No significant change was observed in animal body weight between LCO-treated and control mice during the LCO administration period. No significant differences were observed between (**c**) liver- or (**d**) spleen-weight-to-body-weight ratios between control and LCO-treated mice.

**Figure 5:**
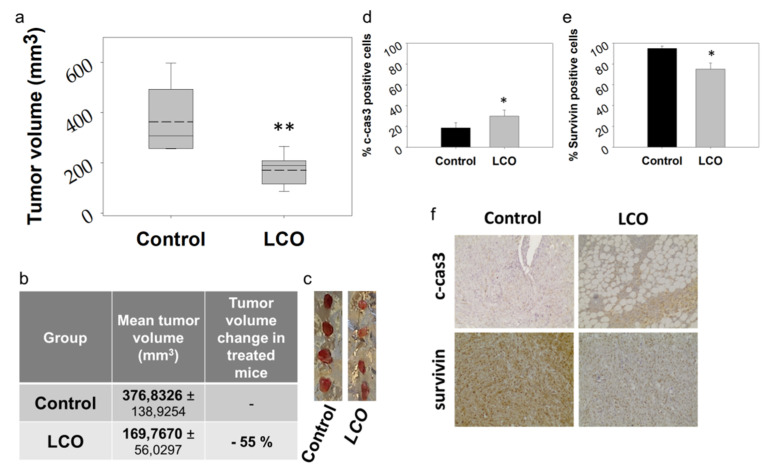
Oral administration of LCO attenuates tumor growth in DA3 breast cancer model in mice. LCO was administered daily to BALB/c mice by oral gavage for 14 days and, on the 12th day, mice were inoculated subcutaneously with DA3 cancer cells (5 × 10^6^ cells per mouse). Seven days later, animals were euthanized and the developed tumors were harvested. A statistically significant reduction of ≈55% in tumor volume (*p* = 0.001, Student’s *t*-test) was observed in LCO-treated animals compared to the control group. (**a**) Graphical representation of tumor volume distribution (solid lines indicate median and dashed lines indicate mean values), (**b**) mean tumor volume, and (**c**) photographic observation of excised tumors. (**d**–**f**) Immunohistochemical detection of (**d**) cleaved caspase-3 and (**e**) survivin in tumor tissue from LCO-treated or control mice. (**f**) Representative images showing the effect of LCO administration on survivin andc-cas3 protein levels. The differences in the expression of cleaved cas-3 or survivin in tumor tissue from treated versus control mice are statistically significant (*p* = 0.023 or *p* = 0.024, respectively), as calculated using the SigmaPlot 11.0 statistical software; (* *p* < 0.05, ** *p* < 0.005).

Oral administration of LCO for 14 days (Figure 4a) induced a statistically significant inhibition in the growth of DA3-tumors in BALB/c mice compared to control animals (Figure 5), while no indications of adverse or toxic effects were observed. Specifically, the daily oral administration of 0.552 g/kg of body weight of LCO in female BALB/c mice for 14 days did not affect animal body weight (Figure 4b) or liver or spleen indexes (Figure 4c,d), which are sensitive indicators for the detection of toxicity in BALB/c mice [54]. Moreover, while animal health was daily monitored for signs of disease or discomfort, no signs of pain or behavioral deviations from normal were detected. Unfortunately, there are no previous studies regarding the in vivo toxicity of the essential oil extracted from *L. citriodora*. Etemad et al. studied the in vivo toxicological profile of the intraperitoneally administered aqueous extract from the plant and reported that the LD_50_ in BALB/c mice is 5 g/kg of body weight [55], a dose nine times lower than the dose used in our experiments. Moreover, several oral studies on citral, which constitutes 43.6% of LCO (Table 1), reported no adverse effects for a dose of up to 1.0 g/kg animal weight in rodents for an administration period of 14 days to 13 weeks [56,57].

Noteworthy, the administration scheme of LCO, described in Figure 4a, induced a 55% inhibition in tumor volume in the DA3 breast adenocarcinoma model. In specific, 7 days post cancer cells’ inoculation, control animals developed tumors of an average size of 377 mm^3^, while LCO-treated animals developed tumors of an average size of 170 mm^3^. To the best of our knowledge, this is the first report on the antitumor potential of orally administered LCO.

### 3.6. LCO Increases Cleaved Caspase 3 Levels and Reduces Protein Expression of Survivin (BIRC5a) in DA3 Tumors

In accordance with our in vitro results (Figure 2), the apoptotic marker cleaved caspase 3 (c-cas3) was elevated in the tumor tissue of LCO-treated animals. In specific, an average of 30% of cells was positive for active caspase 3 in mice that had been receiving LCO compared to 19% in control animals (Figure 5d). These results, along with the elevated pro-apoptotic-to-live cells’ ratios that were detected in vitro in DA3 cells exposed to LCO, indicate that apoptosis might be among the mechanisms initiated by LCO in breast cancer cells. Moreover, protein expression of survivin, a proliferation marker and negative regulator of apoptosis that is highly expressed in most human tumors [58], was reduced by 20% in the tumor tissue of LCO-treated mice (Figure 5e).

## 4. Conclusions

Our results provide compelling evidence for the antitumor potential of LCO against breast cancer. We demonstrated that LCO, as a mixture of various phytochemicals with antioxidant activity, inhibits the proliferation of breast cancer cells in vitro and attenuates tumor growth in vivo in a murine breast adenocarcinoma model. In line with the traditional use of the plant in tea infusion preparations, we decided to use oral exposure as the route of LCO administration. Despite the restricted bioavailability due to pre-systemic metabolism, LCO, as a mixture of phytochemicals, induced a 55% inhibition in tumor volume. Noteworthy, LCO attenuated the migration and proliferation of DA3 breast cancer cells in vitro, partially by inducing apoptotic cell death as evidenced by the Annexin V/PI assay. Moreover, orally administered LCO induced the activation of caspase 3 and downregulated the protein expression of the anti-apoptotic protein survivin in mouse tumor tissue. Taken together, these results confirm the hypothesis that the essential oil extracted from the plant *L. citriodora* exerts antitumor effects against breast adenocarcinoma and highlight the interest of LCO as a source of bioactive compounds with great nutraceutical potential. LCO’s most abundant phytochemical, citral, is a good candidate for further investigation in future studies.

## Figures and Tables

**Table 1:** The 10 most abundant volatile compounds identified in *Lippia citriodora* essential oil (LCO) by GC-MS analysis. Data reproduced from Fitsiou et al. [27].

KRI ^a^	Compounds	% Area	Structure	Formula	MW ^b^ (g/mol)
1246	Geranial (trans-citral)	26.404	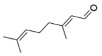	C_10_H_16_O	152.23
1215	Neral (cis-citral)	17.16	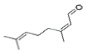	C_10_H_16_O	152.23
1212	Nerol	8.047	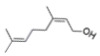	C_10_H_18_O	154.25
1241	Geraniol	5.72	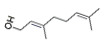	C_10_H_18_O	154.25
1551	Spathulenol	3.279	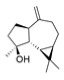	C_15_H_24_O	220.35
1008	1,8-Cineol	3.15	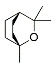	C_10_H_18_O	157.27
954	6-Methyl-5-hepten-2-one	2.278	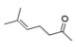	C_8_H_14_O	126.2
1010	Limonene	2.166	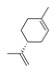	C_10_H_16_	136.23
1464	Ar-curcumene	2.098	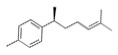	C_15_H_22_	202.33
1473	Bicyclogermacrene	1.75	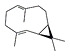	C_15_H_24_	204.35

^a^ KRI: Kovats Retention Indices, ^b^ MW: Molecular Weight.

**Table 2:** IC_50_ values (concentration that causes a 50% inhibition in cell growth) of LCO against DA3 cells determined by the SRB assay after a 24-, 48-, or a 72-h treatment. Data are representative of at least three independent experiments and are presented as mean ± SD.

IC_50_ (24)	IC_50_ (48)	IC_50_ (72)
96.4 ± 8.9 μg/mL	77.8 ± 1.5 μg/mL	70.7 ± 5.5 μg/mL

## Data Availability

Supporting data are available from the authors upon request.
